# Reconstruction of the pelvis and perineum with a free latissimus dorsi myocutaneous flap: a case report

**DOI:** 10.1308/003588412X13373405387537

**Published:** 2012-05

**Authors:** I Kieran, N Nugent, M Ó Riordain, J Kelly

**Affiliations:** ^1^Cork University Hospital,Ireland; ^2^Mercy University Hospital, Cork,Ireland

**Keywords:** Latissimus dorsi, Perineum, Pelvic

## Abstract

Reconstruction of the perineum and pelvic cavity in continuity is an uncommon and difficult challenge. This case describes a 66-year-old man who presented following recurrence of a Dukes’ B rectosigmoid adenocarcinoma that had been treated nine years previously by anterior resection, 5-fluorouracil and radiotherapy. His recurrent disease was treated with radical pelvic exenteration with formation of an end colostomy and urinary ileal conduit.

A post-operative pelvic collection necessitated incisional drainage via the perineum. This resulted in a perineal defect in continuity with the pelvic cavity, neither of which healed in spite of alternate day packing with antiseptic dressings. The perineum and cavity were reconstructed successfully with a microvascular transfer of the latissimus dorsi using the primary gracilis pedicle as recipient donor vessels.

Reconstruction of the perineum is most commonly facilitated using local flap options such as the gracilis, gluteus, rectus abdominis, anterolateral and posterolateral thigh flaps. For smaller defects, the gracilis (a thin strap muscle with little bulk) is often adequate and can facilitate a large paddle of skin in some cases. In the case of larger defects, the rectus abdominis flap is the most commonly employed in gynaecological reconstructions of the perineum. However, formation of a colostomy, ileal conduit or even laparotomy alone can damage the perforating vessels needed for the rectus abdominis flap. In the absence of these flaps to hand, the latissimus dorsi (LD) provides an excellent substitute as well as substantial coverage for these larger defects.

Based on a literature review, it is evident that the use of free LD myocutaneous flaps for perineal and pelvic reconstruction is uncommon. Five articles in the English language were found in the literature and these described seven cases: three pelvic and four perineal reconstructions.

## Case history

Our 66-year-old patient originally had an anterior resection for a Dukes’ B rectosigmoid adenocarcinoma followed by post-operative 5-fluorouracil and radiotherapy. He developed several radiation related complications including severe radiation enterocolitis, small bowel obstruction, ileal stricture and an enterovesical fistula. The latter two required small bowel resection and a diversion procedure respectively.

**Figure 1:**
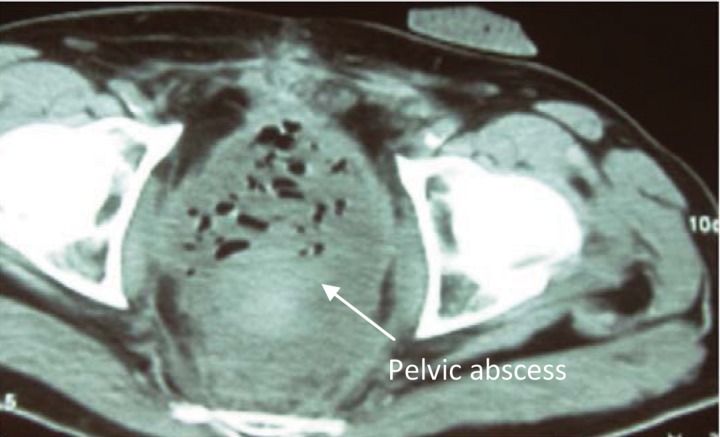
Axial computed tomography of the pelvis showing pelvic abscess

After a disease free period of eight years, the patient presented with a malignant stricture of the neorectum. This necessitated pelvic exenteration consisting of an *en bloc* excision of the neorectum, the rectal stump, the mesorectum, the bladder with a segment of diverted small bowel and the prostate. Both the end colostomy and ileal conduit were fashioned. His post-operative recovery was complicated by the development of a pelvic haematoma requiring incisional drainage via the perineum (Fig 1).

Following drainage, the pelvic cavity was packed with saline soaked dressings in anticipation of healing via secondary intention. Subsequent alternate day packing was undertaken under general anaesthesia. However, little healing was noted over the course of these dressing changes. A trial of negative pressure wound therapy also failed to reduce the cavity size, which extended from the pubic symphysis to the coccyx internally and communicated with a perineal defect measuring approximately 10cm × 8cm. Within the abdomen, due to the previous radiation, the remaining bowel and omentum remained adherent at the level of the pelvic brim. Owing to this adherent bowel and the bony confines of the pelvis, secondary obliteration of the cavity could not occur. It was evident after nine months that the cavity walls were becoming re-epithelialised without any reduction in the cavity size.

As mentioned above, gracilis, rectus abdominis, bilateral gluteus maximus, anterolateral and posterolateral thigh flaps are the more commonly used flaps for closure of perineal defects. Given the substantial size of this defect combined with its continuity with an empty pelvic cavity, a gracilis muscle flap provided insufficient volume. Furthermore, bilateral stomas precluded the use of the rectus abdominis muscles and both gluteal muscles were too atrophied for use. An anterolateral or posterolateral thigh flap would have lacked the muscle bulk required to fill the interior defect and a myocutaneous flap was preferable in this setting due to the chronicity and previous contamination of the wound. A free transfer of tissue from elsewhere was the only remaining option.

**Figure 2:**
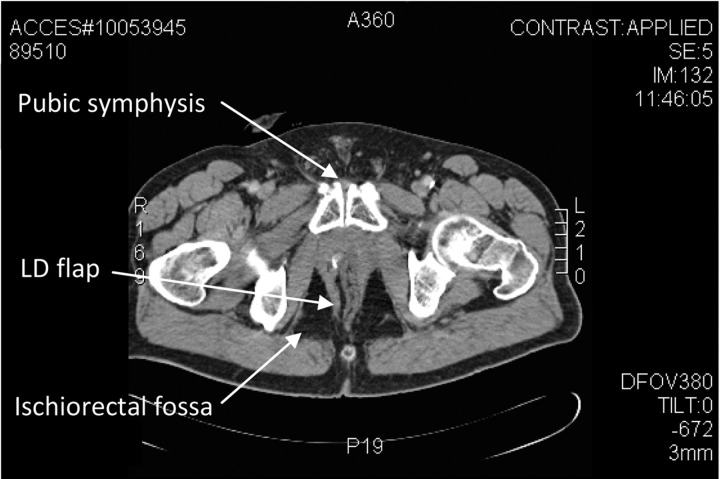
Axial computed tomography of the pelvis following latissimus dorsi (LD) reconstruction of perineum and pelvis

**Figure 3:**
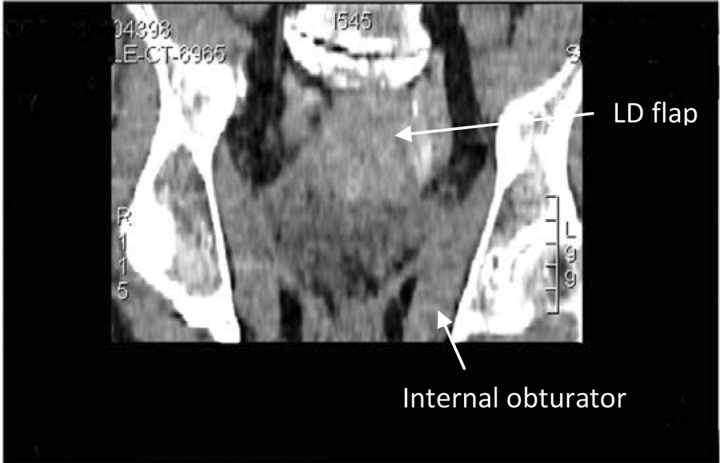
Coronal computed tomography of the pelvis following latissimus dorsi (LD) reconstruction of perineum and pelvis

Initial steps included preparation of the cavity, which had become lined with epithelium. With the patient in the lithotomy position, careful de-epithelialisation of the pelvic walls and sections of the small bowel at the upper end of the cavity was performed using gentle curetting and argon plasma coagulation. Turning the patient on to his left lateral side, the LD myocutaneous flap was raised from the right side. Following repositioning into lithotomy, microvascular transplantation of the LD was performed, anastomosing the thoracodorsal vessels to the primary gracilis pedicle, a branch of the medial femoral circumflex system. The thoracodorsal pedicle was tunnelled subcutaneously for a distance of 5cm along the medial thigh and the muscle was inserted through the perineal defect, filling the pelvic cavity (Figs 2 and 3). The overlying skin paddle facilitated primary closure of the perineal defect.

The patient continues to do well four years post-operatively and recent surveillance computed tomography (CT) shows no evidence of tumour recurrence or a keratinoma.

## Discussion

Difficulties encountered particular to this case were a persistent and significant volume of dead space in the pelvic cavity, the continuity of this cavity with a large perineal defect and a progressive re-epithelialisation of the cavity walls within. The process of cavity wall re-epithelialisation has the potential to develop a keratinoma if allowed to proliferate. However, the exact incidence of this has not been reported in the literature. A keratinoma can grow slowly over time and, if it becomes infected, it is a potential source of overwhelming sepsis. Treatment is by excision. Surveillance CT will be required in this case to monitor for epithelial regrowth as well as for tumour recurrence.

The large, pelvic dead space encountered was mainly contributed to by previous radiation enterocolitis with the resultant adherence of small bowel and omentum to the abdominal wall. This lack of descent prevented the required obliteration of the cavity. A combination of various disease states of the perineum, its adjacent orifices and dressing challenges make perineal healing an arduous task. This is further compounded when a perineal defect is in continuity with empty pelvic cavity, which is at risk of haematoma and seroma development.

Reconstruction of the perineum and pelvis using microvascular transfer of the LD is a relatively recent development and is less commonly opted for if more local myocutaneous options are available. This is supported by a review of the literature, which found five articles (in English) describing three pelvic and four perineal reconstructions using this free flap.

Kraybill *et al* described its use in an early case of pelvic reconstruction in 1982.^1^ It was a case similar to the one presented in this report where microvascular transfer of the LD was used for the reconstruction of the pelvis and perineum following an abdominoperineal resection (APR) that had been complicated by a pelvic abscess and necrosis. Hurst *et al* reported a perineal reconstruction for a complicated perineal wound following a proctectomy for Crohn’s disease.^2^ Petrie *et al* demonstrated a significant success rate of 18 cases of flap closure following APR; 3 of these were microvascular transfer of the LD.^3^ More recently, the same authors reported a further case using an inferior mesenteric artery stump as the donor vessel.^4^ Lin *et al* described the use of this flap in the reconstruction of a perianal wound in continuity with precoccygeal dead space following radiation associated infection after APR.^5^ Recipient donor vessels described to receive the LD transfer in these cases included the femoral, inferior epigastric, inferior mesenteric and gluteal arteries.

## Conclusions

The preferred flaps for the reconstruction of the pelvis and perineum include the gracilis, bilateral gluteus maximus, rectus abdominis, anterolateral and posterolateral thigh flaps. In our case, due to the inaccessibility of these more commonly employed flaps, the LD free flap proved an excellent substitute. Together with a pedicle length of 13cm, an approximate volume of 300cm^3^ and using the gracilis pedicle as the donor vessel, it provided an excellent size match for this defect.
